# Large-scale genome-wide association analyses identify novel genetic loci and mechanisms in hypertrophic cardiomyopathy

**DOI:** 10.1038/s41588-025-02087-4

**Published:** 2025-02-18

**Authors:** Rafik Tadros, Sean L. Zheng, Christopher Grace, Paloma Jordà, Catherine Francis, Dominique M. West, Sean J. Jurgens, Kate L. Thomson, Andrew R. Harper, Elizabeth Ormondroyd, Xiao Xu, Pantazis I. Theotokis, Rachel J. Buchan, Kathryn A. McGurk, Francesco Mazzarotto, Beatrice Boschi, Elisabetta Pelo, Michael Lee, Michela Noseda, Amanda Varnava, Alexa M. C. Vermeer, Roddy Walsh, Ahmad S. Amin, Marjon A. van Slegtenhorst, Nicole M. Roslin, Lisa J. Strug, Erika Salvi, Chiara Lanzani, Antonio de Marvao, Hypergenes InterOmics Collaborators, Daniele Cusi, Paolo Manunta, Lorena Citterio, Nicola Glorioso, Jason D. Roberts, Maxime Tremblay-Gravel, Genevieve Giraldeau, Julia Cadrin-Tourigny, Philippe L. L’Allier, Patrick Garceau, Mario Talajic, Sarah A. Gagliano Taliun, Yigal M. Pinto, Harry Rakowski, Antonis Pantazis, Wenjia Bai, John Baksi, Brian P. Halliday, Sanjay K. Prasad, Paul J. R. Barton, Declan P. O’Regan, Stuart A. Cook, Rudolf A. de Boer, Imke Christiaans, Michelle Michels, Christopher M. Kramer, Carolyn Y. Ho, Stefan Neubauer, HCMR Investigators, Sanjay K. Prasad, Michelle Michels, Christopher M. Kramer, Carolyn Y. Ho, Stefan Neubauer, Theodore Abraham, Lisa Anderson, Florian Andre, Evan Appelbaum, Camillo Autore, Lauren Baldassarre, Colin Berry, Elena Biagini, William Bradlow, Chiara Bucciarelli-Ducci, Amedeo Chiribiri, Lubna Choudhury, Andrew Crean, Dana Dawson, Milind Desai, Patrice Desvigne-Nickens, John DiMarco, Eleanor Elstein, Andrew Flett, Matthias Friedrich, Eli Gelfand, Nancy Geller, Tjeerd Germans, Jeffrey Geske, Allison Hays, Stephen B. Heitner, Adam Helms, Daniel Jacoby, Dong-Yun Kim, Bette Kim, Han Kim, Paul Kolm, Raymond Kwong, Eric Larose, Christopher Madias, Masliza Mahmod, Heiko Mahrholdt, Martin Maron, Ahmad Masri, Gerry McCann, Saidi Mohiddin, Francois-Pierre Mongeon, Sherif Nagueh, David Newby, Angus Nightingale, Anjali Owens, Sven Plein, Betty Raman, Ornella Rimoldi, Michael Salerno, Jeanette Schulz-Menger, Sanjay Sharma, Mark Sherrid, Albert van Rossum, Jonathan Weinsaft, William Weintraub, James White, Eric Williamson, Anna Woo, Iacopo Olivotto, Hugh Watkins, Paul M. Matthews, Arthur A. M. Wilde, Jean-Claude Tardif, Iacopo Olivotto, Arnon Adler, Anuj Goel, James S. Ware, Connie R. Bezzina, Hugh Watkins

**Affiliations:** 1 Cardiovascular Genetics Centre and Research Centre, Montreal Heart Institute, Montreal, Quebec Canada; 2 Faculty of Medicine, Université de Montréal, Montreal, Quebec Canada; 3 Department of Experimental Cardiology, Amsterdam Cardiovascular Sciences, University of Amsterdam, Amsterdam UMC, Amsterdam, the Netherlands; 4 National Heart and Lung Institute, Imperial College London, London, UK; 5 MRC Laboratory of Medical Sciences, Imperial College London, London, UK; 6 Royal Brompton and Harefield Hospitals, Guy’s and St. Thomas’ NHS Foundation Trust, London, UK; 7 Division of Cardiovascular Medicine, Radcliffe Department of Medicine, University of Oxford, John Radcliffe Hospital, Oxford, UK; 8 Cardiovascular Disease Initiative, Broad Institute of MIT and Harvard, Cambridge, MA USA; 9 Oxford Genetics Laboratories, Churchill Hospital, Oxford, UK; 10 Department of Molecular and Translational Medicine, University of Brescia, Brescia, Italy; 11 Genetics Unit, Careggi University Hospital, Florence, Italy; 12 Imperial College Healthcare NHS Trust, Imperial College London, London, UK; 13 Department of Clinical Genetics, University of Amsterdam, Amsterdam UMC, Amsterdam, the Netherlands; 14 European Reference Network for Rare and Low Prevalence Complex Diseases of the Heart (ERN GUARD-HEART), Amsterdam, the Netherlands; 15 Department of Clinical Cardiology, Amsterdam Cardiovascular Sciences, University of Amsterdam, Amsterdam UMC, Amsterdam, the Netherlands; 16 Department of Clinical Genetics, Erasmus Medical Center, University Medical Center Rotterdam, Rotterdam, the Netherlands; 17 Program in Genetics and Genome Biology and The Centre for Applied Genomics, The Hospital for Sick Children, Toronto, Ontario Canada; 18 Departments of Statistical Sciences and Computer Science, University of Toronto, Toronto, Ontario Canada; 19 Division of Biostatistics, Dalla Lana School of Public Health, University of Toronto, Toronto, Ontario Canada; 20 Neuroalgology Unit, Fondazione IRCCS Istituto Neurologico ‘Carlo Besta’, Milan, Italy; 21 Genomics of Renal Diseases and Hypertension Unit and Nephrology Operative Unit, IRCCS San Raffaele Hospital, Milan, Italy; 22 Vita-Salute San Raffaele University, Milan, Italy; 23 King’s College London, London, UK; 24 Department of Medicine, Section of Cardiac Electrophysiology, Division of Cardiology, Western University, London, Ontario Canada; 25 Division of Cardiology, Peter Munk Cardiac Centre, University Health Network, Toronto, Ontario Canada; 26 Department of Computing, Imperial College London, London, UK; 27 Department of Brain Sciences, Imperial College London, London, UK; 28 Data Science Institute, Imperial College London, London, UK; 29 National Heart Centre, Singapore, Singapore; 30 Duke-National University of Singapore Medical School, Singapore, Singapore; 31 Department of Cardiology, Thorax Center, Cardiovascular Institute, Erasmus Medical Center, Rotterdam, the Netherlands; 32 Department of Genetics, University of Groningen, University Medical Center Groningen, Groningen, the Netherlands; 33 Department of Medicine, Cardiovascular Division, University of Virginia Health, Charlottesville, VA USA; 34 Cardiovascular Division, Brigham and Women’s Hospital, Boston, MA USA; 35 Radcliffe Department of Medicine, University of Oxford, Division of Cardiovascular Medicine, NIHR Oxford Biomedical Research Centre, Oxford, UK; 36 UK Dementia Research Institute, Imperial College London, London, UK; 37 ECGen, Cardiogenetics Focus Group of EHRA, Biot, France; 38 Meyer Children’s Hospital IRCCS, Florence, Italy; 39 Department of Medicine, University of Toronto, Toronto, Ontario Canada; 40 Program in Medical and Population Genetics, Broad Institute of MIT and Harvard, Cambridge, MA USA; 41 Institute of Biomedical Technologies Milano National Research Council of Italy (CNR), Milan, Italy; 42 Genomics of Renal Diseases and Hypertension Unit, Istituto di Ricovero e Cura a Carattere Scientifico IRCCS San Raffaele Scientific Institute, Vita-Salute San Raffaele University, Milan, Italy; 43 Department of Clinical and Experimental Medicine, Hypertension and Related Diseases Centre, University of Sassari, Sassari, Italy; 44 Johns Hopkins University, Baltimore, MD USA; 45 St-George’s University of London, London, UK; 46 University Hospital Heidelberg, Heidelberg, Germany; 47 Beth Israel Deaconess Medical Center, Boston, MA USA; 48 Sapienza University of Rome, Rome, Italy; 49 Yale School of Medicine, New Haven, CT USA; 50 University of Glasgow, Glasgow, Scotland; 51 University of Bologna, Bologna, Italy; 52 University Hospital Birmingham, Birmingham, UK; 53 University of Bristol, Bristol, UK; 54 Northwestern University, Chicago, IL USA; 55 University of Aberdeen, Aberdeen, UK; 56 Cleveland Clinic, Cleveland, OH USA; 57 National Heart Lung & Blood Institute, Bethesda, MD USA; 58 McGill University, Montreal, Quebec Canada; 59 University Hospital Southampton, Southampton, UK; 60 Amsterdam Cardiovascular Sciences, Vrije Universiteit Amsterdam, Amsterdam UMC, Amsterdam, the Netherlands; 61 Mayo Clinic, Rochester, MN USA; 62 Oregon Health & Science University, Portland, OR USA; 63 University of Michigan, Ann Arbor, MI USA; 64 Icahn School of Medicine at Mount Sinai, New York, NY USA; 65 Duke University, Durham, NC USA; 66 MedStar Health Research Institute, Washington, DC USA; 67 Quebec Heart and Lung Institute, Quebec City, Quebec Canada; 68 Tufts Medical Center, Boston, MA USA; 69 Robert-Bosch-Krankenhaus, Stuttgart, Germany; 70 University of Leicester, Leicester, UK; 71 Barts Heart Centre, London, UK; 72 Methodist DeBakey Heart and Vascular Center, Houston, TX USA; 73 University of Edinburgh, Edinburgh, UK; 74 Perelman School of Medicine, University of Pennsylvania, Philadelphia, PA USA; 75 University of Leeds, Leeds, UK; 76 San Raffaele Hospital, Milan, Italy; 77 Charité - Universitätsmedizin Berlin, Berlin, Germany; 78 New York University Langone Medical Center, New York, NY USA; 79 Division of Cardiology, Weill Cornell Medicine, New York, NY USA; 80 Libin Cardiovascular Institute, University of Calgary, Calgary, Alberta Canada

**Keywords:** Cardiomyopathies, Genome-wide association studies

## Abstract

Hypertrophic cardiomyopathy (HCM) is an important cause of morbidity and mortality with both monogenic and polygenic components. Here, we report results from a large genome-wide association study and multitrait analysis including 5,900 HCM cases, 68,359 controls and 36,083 UK Biobank participants with cardiac magnetic resonance imaging. We identified 70 loci (50 novel) associated with HCM and 62 loci (20 novel) associated with relevant left ventricular traits. Among the prioritized genes in the HCM loci, we identify a novel HCM disease gene, *SVIL*, which encodes the actin-binding protein supervillin, showing that rare truncating *SVIL* variants confer a roughly tenfold increased risk of HCM. Mendelian randomization analyses support a causal role of increased left ventricular contractility in both obstructive and nonobstructive forms of HCM, suggesting common disease mechanisms and anticipating shared response to therapy. Taken together, these findings increase our understanding of the genetic basis of HCM, with potential implications for disease management.

## Abstract (web summary)

Genome-wide and multitrait analyses identify novel loci associated with hypertrophic cardiomyopathy and relevant left ventricular traits. Gene-level burden analyses show that rare truncating *SVIL* variants are associated with high risk of hypertrophic cardiomyopathy.

## Main

HCM is a disease of the cardiac muscle characterized by thickening of the left ventricular (LV) wall with or without obstruction of flow (obstructive, oHCM; nonobstructive, nHCM). HCM is associated with an increased risk of arrhythmia, heart failure, stroke and sudden death. Previously viewed as a Mendelian disease with rare pathogenic variants in cardiac sarcomere genes identified in ~35% of cases (HCM_SARC+_), HCM is now known to have complex and diverse genetic architectures^1^. Previous studies have established that common genetic variants underlie a large portion of disease heritability in HCM not caused by rare pathogenic variants (sarcomere-negative (HCM_SARC−_)) and partly explain the variable expressivity in HCM patients carrying pathogenic variants (sarcomere-positive (HCM_SARC+_)), but such studies had limited power to identify a large number of significant loci^2,3^.

**Fig. 1:**
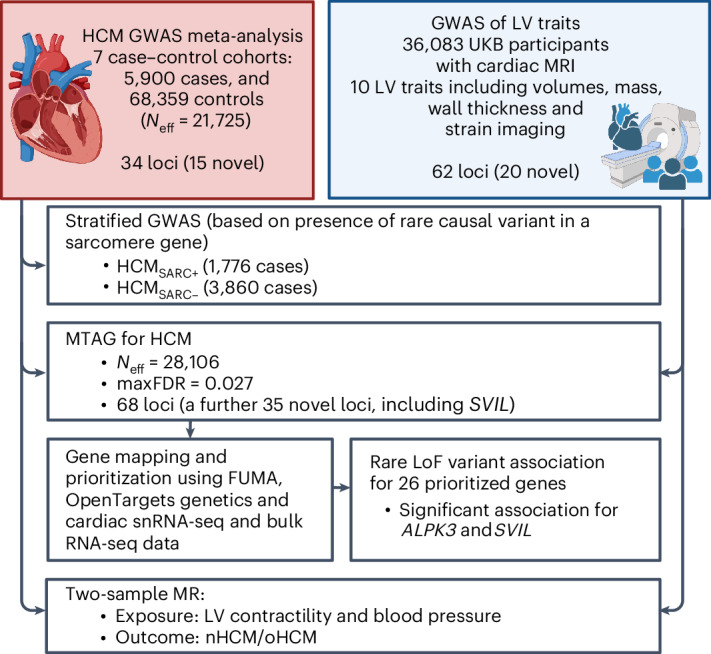
Study flowchart. Flowchart of meta-analysis of seven case–control HCM GWAS datasets, GWAS of LV traits and downstream analyses. Created using BioRender.com.

We report a meta-analysis of seven case–control HCM genome-wide association study (GWAS) datasets comprising a total of 5,900 HCM cases, 68,359 controls and 9,492,702 variants with a minor allele frequency (MAF) > 1% (Supplementary Table 1; see study flowchart in Fig. 1). We identified 34 loci significantly associated with HCM (at *P* < 5 × 10^−8^), of which 15 were novel (Table 1 and Supplementary Figs. 1 and 2a). Stratified analyses in HCM_SARC+_ (1,776 cases) and HCM_SARC−_ (3,860 cases) identified a further five loci (Table 1, Supplementary Fig. 2b and Supplementary Table 2). Using conditional analysis^4^, we identified more independent associations with HCM, HCM_SARC+_, and HCM_SARC−_ with a false discovery rate (FDR) < 1% (Supplementary Table 3). We estimated the heritability of HCM attributable to common genetic variation (*h*^2^_SNP_) in the all-comer analysis to be 0.17 ± 0.02 using linkage disequilibrium (LD) score regression (LDSC)^5^, and found higher estimates (0.25 ± 0.02) using genome-based restricted maximum likelihood (GREML)^6^, with higher *h*^2^_SNP_ in HCM_SARC−_ (0.29 ± 0.02) compared with HCM_SARC+_ (0.16 ± 0.04) (Supplementary Table 4).

**Table 1:** Lead variants from the HCM GWAS

Lead SNP	GRCh37	EA/NEA	EAF	OR (95% CI)	*P*	*Q*	Locus name	GWS in HCM_SARC+_	GWS in HCM_SARC−_
**Genome-wide significant loci from all HCM meta-analysis**									
rs2234962	10:121429633	C/T	0.21	1.45 (1.38–1.52)	1.39 × 10^−49^	0.36	*BAG3* (missense)	•	•
rs2644262	18:34223566	C/T	0.29	1.38 (1.32–1.45)	1.79 × 10^−43^	0.43	*FHOD3/TPGS2*	•	•
rs78310129	11:56793878	T/C	0.01	3.53 (2.92–4.27)	9.79 × 10^−39^	1.59E-09	*MYBPC3*	•	
rs1048302	1:16340879	T/G	0.33	1.28 (1.23–1.34)	8.47 × 10^−30^	0.59	*HSPB7*		•
rs2070458*	22:24159307	A/T	0.22	1.30 (1.24–1.37)	5.93 × 10^−25^	0.09	*VPREB3/SMARCB1*		•
rs3176326*	6:36647289	A/G	0.21	1.30 (1.24–1.37)	3.18 × 10^−24^	0.14	*CDKN1A*		•
rs12212795	6:118654308	C/G	0.05	1.51 (1.39–1.65)	4.76 × 10^−22^	0.33	*SLC35F1/PLN*		•
rs4577128*	17:64308473	C/T	0.57	1.23 (1.18–1.29)	3.26 × 10^−21^	0.97	*PRKCA*		•
rs393838	17:43705756	C/G	0.23	1.26 (1.20–1.32)	5.02 × 10^−21^	0.91	*CRHR1/MAPT*		•
rs8033459*	15:85253258	T/C	0.46	1.20 (1.15–1.25)	7.04 × 10^−18^	0.66	*ALPK3/NMB*	•	•
rs11196085*	10:114505037	C/T	0.28	1.22 (1.16–1.28)	1.85 × 10^−17^	0.75	*VTI1A/TCF7L2*		•
rs7301677	12:115381147	C/T	0.74	1.22 (1.16–1.29)	7.01 × 10^−16^	0.27	*TBX3*		•
rs2177843*	10:75409877	T/C	0.16	1.26 (1.19–1.34)	2.80 × 10^−15^	0.91	*MYOZ1/SYNPO2L*		•
rs41306688	13:114078558	C/A	0.03	1.60 (1.42–1.80)	3.04 × 10^−15^	0.22	*ADPRHL1* (missense)		•
rs2191445*	5:57011469	T/A	0.80	1.23 (1.17–1.30)	8.22 × 10^−14^	0.37	*ACTBL2*		•
rs4894803*	3:171800256	G/A	0.41	1.18 (1.13–1.24)	2.19 × 10^−13^	0.63	*FNDC3B*		•
rs13061705	3:14291129	C/T	0.69	1.19 (1.13–1.25)	5.67 × 10^−13^	0.68	*SLC6A6/LSM3*		•
**rs13021775**	**2:37059557**	**C/G**	**0.50**	**1.17 (1.12–1.23)**	**5.98** **×** **10**^**−13**^	0.50	***STRN***		•
**rs8006225**	**14:95219657**	**G/T**	**0.83**	**1.22 (1.15–1.30)**	**2.64** **×** **10**^**−11**^	0.15	***GSC***		•
rs10052399*	5:138668504	T/C	0.27	1.18 (1.12–1.24)	3.99 × 10^−11^	0.03	*SPATA24*		
rs66520020*	7:128438284	T/C	0.16	1.21 (1.14–1.28)	5.87 × 10^−11^	0.96	*CCDC136/FLNC*		
**rs12460541**	**19:46312077**	**G/A**	**0.66**	**1.16 (1.11–1.21)**	**6.01** **×** **10**^**−11**^	0.13	***DMPK/SYMPK***		
**rs7461129**	**8:125861374**	**T/C**	**0.31**	**1.16 (1.11–1.21)**	**8.19** **×** **10**^**−11**^	0.87	***MTSS1***		
**rs56005624**	**2:179774634**	**G/T**	**0.14**	**1.21 (1.14–1.28)**	**8.31** **×** **10**^**−11**^	0.62	***CCDC141/SESTD1***		•
**rs7824244**	**8:21802432**	**A/G**	**0.14**	**1.22 (1.14–1.29)**	**2.39** **×** **10**^**−10**^	0.34	***XPO7***	•	
**rs12270374**	**11:14375079**	**C/T**	**0.36**	**1.14 (1.09–1.20)**	**6.85** **×** **10**^**−10**^	0.92	***RRAS2/COPB1***		
**rs62222424**	**21:30530131**	**G/A**	**0.93**	**1.32 (1.20–1.44)**	**1.21** **×** **10**^**−9**^	0.69	***CCT8***		
**rs11687178**	**2:11584197**	**C/A**	**0.65**	**1.14 (1.09–1.19)**	**7.70** **×** **10**^**−9**^	0.26	***E2F6/ROCK2***		
**rs9320939**	**6:123818871**	**A/G**	**0.49**	**1.13 (1.08–1.18)**	**1.04** **×** **10**^**−8**^	0.04	***TRDN/HEY2***		•
**rs2540277**	**2:103426177**	**C/T**	**0.94**	**1.32 (1.19–1.45)**	**2.31** **×** **10**^**−8**^	0.84	***TMEM182/MFSD9***		
**rs6566955**	**18:55922789**	**G/A**	**0.31**	**1.14 (1.08–1.19)**	**2.93** **×** **10**^**−8**^	0.16	***NEDD4L***		
**rs13004994**	**2:220406239**	**T/G**	**0.46**	**1.13 (1.08–1.18)**	**3.02** **×** **10**^**−8**^	0.12	***CHPF***		
**rs2645210**	**10:4098453**	**A/G**	**0.19**	**1.16 (1.10–1.23)**	**3.94** **×** **10**^**−8**^	0.52	***KLF6/AKR1E2***		
**rs113907726**	**14:53316867**	**G/T**	**0.19**	**1.16 (1.10–1.22)**	**4.10** **×** **10**^**−8**^	0.27	***FERMT2/ERO1A***		
**Additional loci discovered in HCM**_**SARC+**_ **or HCM**_**SARC−**_									
rs9311485	3:52987645	T/G	0.25	1.13 (1.08–1.19)	1.86 × 10^−7^	0.09	*ITIH3/SFMBT1*		•
rs77963625	12:46446897	C/T	0.03	1.38 (1.22–1.57)	2.97 × 10^−7^	0.24	*SCAF11*		•
rs846111	1:6279370	G/C	0.73	1.14 (1.08–1.20)	6.32 × 10^−7^	0.52	*RNF207* (missense)		•
rs58747679	12:26348304	T/C	0.71	1.12 (1.07–1.18)	1.30 × 10^−6^	0.15	*SSPN*		•
rs112787369	14:68252852	T/A	0.04	1.21 (1.08–1.35)	6.04× 10^−4^	0.62	*ZYVE26* (missense)	•	

All reported summary statistics refer to the all HCM case–control meta-analysis results, including for loci identified only in the HCM_SARC+_ and HCM_SARC−_ stratified analyses. The table is sorted in increasing order of the all-comer *P* values. Novel loci are shown in bold. An asterisk marks loci that reached significance in a previous multitrait analysis of GWAS (MTAG)^3^ and now reach significance in the present GWAS. Locus naming was performed primarily by OpenTargets^13^, also considering functional mapping and annotation of GWAS (FUMA)^14^ mapping, and previous rare variant associations with HCM^26^. Dots indicate the presence of GWS. EA/NEA, effect and noneffect alleles; EAF, effect allele frequency; GWS, genome-wide significance (*P* ≤ 5 × 10^−8^); *Q*, Cochrane’s heterogeneity test *P* value.

**Fig. 2:**
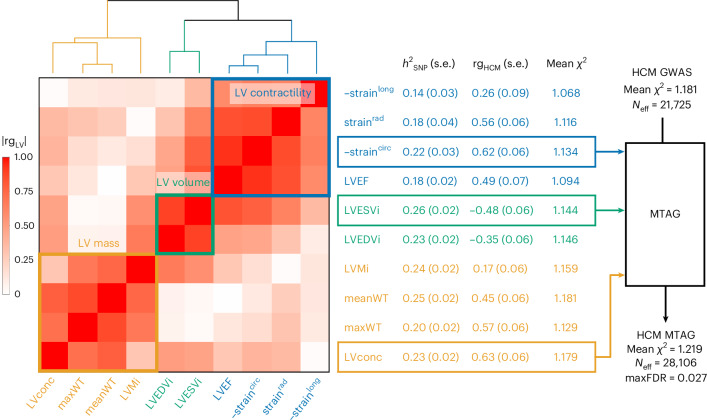
Genetic correlation of LV traits and HCM and use of MTAG to empower locus discovery. Pairwise genetic correlation between LV traits shown in heatmap as absolute values (|rg_LV_|) ranging from 0 (white) to 1 (red). LV traits are sorted into three clusters based on |rg_LV_| along the *x* and *y* axes using Euclidean distance and complete hierarchical clustering: LV contractility (blue), volume (bluish green) and mass (orange) (see dendrogram on top). The table in the middle shows the individual LV trait *h*^2^_SNP_ and genetic correlation with HCM (rg_HCM_), with corresponding s.e. The trait with the strongest correlation (based on rg_HCM_) in each of the three clusters was carried forward for MTAG to empower locus discovery in HCM. MTAG resulted in an increase in *N*_eff_, based on number of cases and controls and increase in mean *χ*^2^ statistic from 21,725 to 28,106, with an estimated maxFDR of 0.027. Since strain^circ^ and strain^long^ are negative values where increasingly negative values reflect increased contractility, we show −strain^circ^ and −strain^long^ to facilitate interpretation of rg_HCM_ sign. Full rg_LV_ and rg_HCM_ results are shown in Supplementary Table 7.

**Fig. 3:**
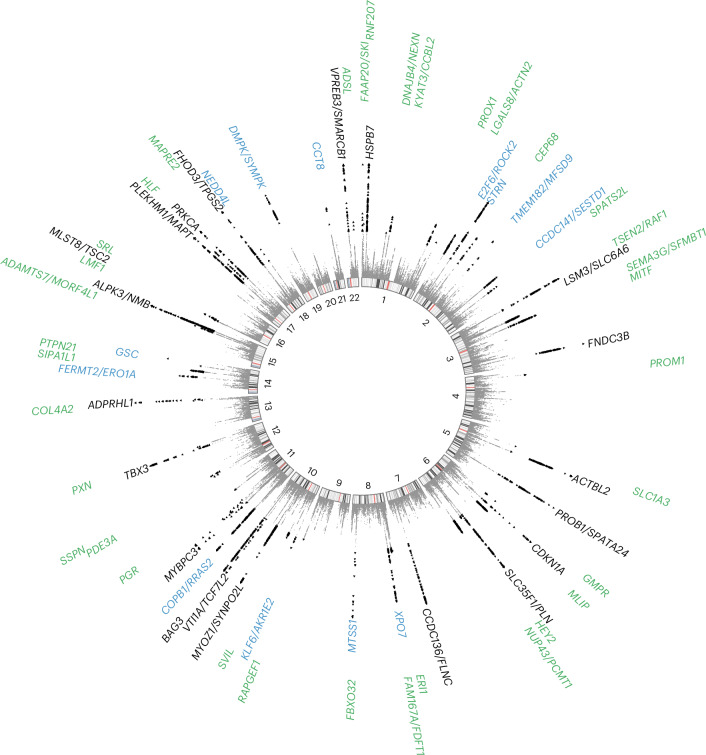
Circular Manhattan plot of HCM summary statistics from MTAG analysis. Previously published loci are identified in black (*n* = 20), novel loci discovered by single-trait all-comer GWAS meta-analysis are identified in blue (*n* = 13) and other novel loci from MTAG are identified in green (*n* = 35). Two other loci reaching GWAS significance threshold in the single-trait HCM GWAS meta-analysis but not reaching significance in MTAG are not shown (mapped to *TRDN*/*HEY2* and *CHPF*; Table 1). *P* values are not corrected for multiple testing and correspond to the HCM MTAG including the fixed-effects meta-analysis of seven HCM case–control GWAS and three LV traits (Fig. 2). Significant variants with *P* < 5 × 10^−8^ are shown as black triangles. Results with *P* < 1 × 10^−15^ are assigned *P* = 1 × 10^−15^. Locus naming was performed primarily by OpenTargets gene prioritization considering FUMA and previous gene association with Mendelian HCM. See Supplementary Table 8 for loci details.

To further maximize HCM locus discovery, we performed a multitrait analysis of GWAS (MTAG)^7^ (Fig. 2). We first completed a GWAS of ten cardiomyopathy-relevant LV traits in 36,083 participants of the UK Biobank (UKB), using machine learning assessment of cardiac magnetic resonance (CMR) imaging^8^ for LV volumes, wall thickness and myocardial strain (Supplementary Table 5 and Supplementary Figs. 3–12). We discovered 62 loci associated with LV traits (20 novel) (Supplementary Table 6). LDSC analyses^9^ demonstrated high genetic correlations (rg) between LV traits within three clusters (contractility, volume and mass) and with HCM (Fig. 2 and Supplementary Table 7). Leveraging such correlations, we performed an HCM MTAG by including the three LV traits most correlated with HCM (one trait from each cluster), namely global circumferential strain (contractility cluster; rg = −0.62), LV end-systolic volume (volume cluster; rg = −0.48) and the ratio of LV mass to end-diastolic volume (mass cluster; rg = 0.63). MTAG resulted in a substantial increase in mean *χ*^2^ equivalent to an increase in effective sample size (*N*_eff_) of the HCM GWAS of ~29% (from 21,725 to 28,106), with an estimated upper bound of the false discovery rate (maxFDR)^7^ of 0.027. Effect estimates derived from MTAG were strongly correlated with those from GWAS (Supplementary Fig. 13). MTAG resulted in a substantial step up in loci discovered, identifying a total of 68 loci associated with HCM at *P* < 5 × 10^−8^, including 48 that have not been published previously (13 novel loci also identified in the single-trait HCM GWAS, and 35 additional novel loci from MTAG) (Fig. 3, Supplementary Table 8 and Supplementary Fig. 14). Two of the 34 loci reaching genome-wide significance in the HCM GWAS were not significant in MTAG (loci mapped to *TRDN*/*HEY2* and *CHPF*). The total number of loci identified in GWAS or MTAG is therefore 70 (50 novel). Although it was not possible to test for replication for the 35 novel MTAG loci, a previous study strongly supports the robustness of the HCM-LV traits MTAG approach, whereby all ten HCM loci uncovered using MTAG in this previous study were independently validated^3^, and all reach *P* < 5 × 10^−8^ in the present GWAS.

MAGMA^10^ gene set analysis identified several significant gene sets linked to muscle, contractility and sarcomeric function (Supplementary Table 9), and tissue expression analysis pointed to cardiac tissue (LV and atrial appendage (AA)) (Supplementary Table 10). Within cardiac tissue, we further explored the contribution of specific cell types in HCM by leveraging available single-nuclei RNA sequencing (snRNA-seq) data from donor human hearts^11^. Using sc-linker^12^, we identified significant enrichment of heritability in cardiomyocyte and adipocyte cell types (cardiomyocyte, FDR-adjusted *P* = 1.8 × 10^−6^; adipocyte, FDR-adjusted *P* = 3.0 × 10^−3^) and cell states (Supplementary Fig. 15).

**Fig. 4:**
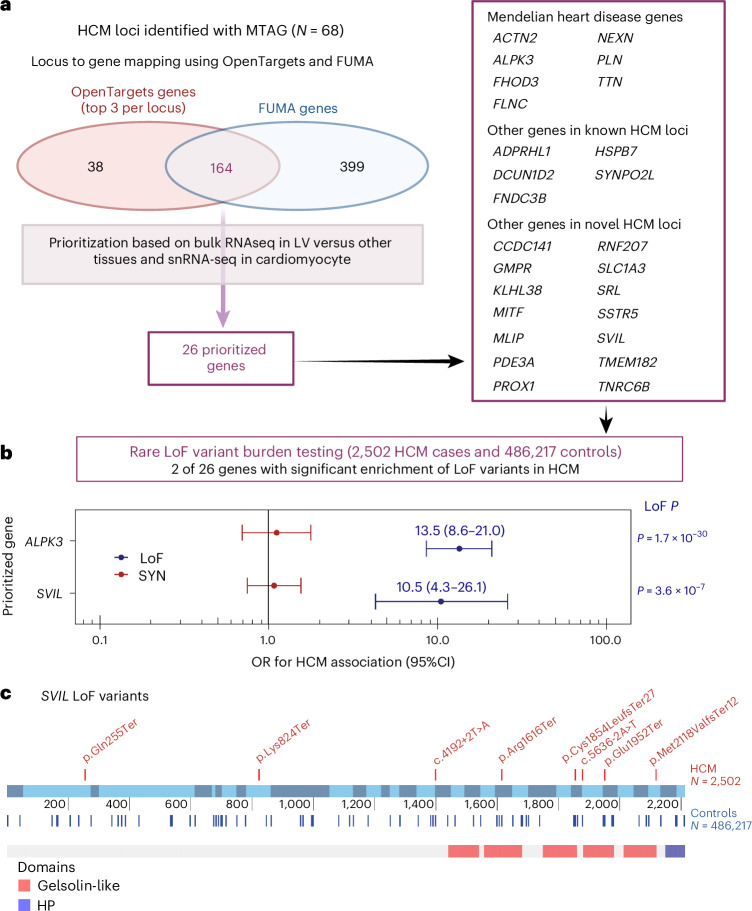
HCM locus-to-gene mapping, prioritization and rare LoF association testing identifies *SVIL* as a new HCM disease gene. **a**, HCM locus-to-gene mapping and prioritization based on cardiac expression. Locus-to-gene mapping was done using the OpenTargets^13^ V2G pipeline (release of 12 October 2022) for all 68 lead variants at the HCM MTAG loci and using FUMA^14^ for the HCM MTAG summary statistics (see Methods for detailed parameters). Of 164 genes mapped using both FUMA and OpenTargets (top 3 genes per locus), 26 were prioritized because of either high specificity of LV expression using the bulk RNA-seq data of the GTEx project^38^ release v.8 and/or high expression in cardiomyocytes using snRNA-seq data^15^. See Methods and Supplementary Tables 12 and 13 for details. **b**, Rare (MAF < 10^−4^) LoF variant association analyses with HCM versus controls performed for all 26 genes using sequencing data in up to 2,502 unrelated HCM cases and 486,217 controls from four datasets followed by IVW meta-analysis. Association of rare synonymous (SYN) variants was also performed as a negative control. Results shown restricted to two genes (*ALPK3* and *SVIL*) reaching the Bonferroni-corrected threshold of *P* < 0.0019 (0.05/26) in the IVW meta-analysis. Filled circles and error bars represent the OR and their 95% CI, respectively, from the meta-analysis for LoF (blue) and SYN (red). *P* values shown are not corrected for multiple testing. Full results appear in Supplementary Table 16. **c**, Schematic of the rare LoF *SVIL* variants in HCM cases (top) and controls (bottom) along the linear structure of *SVIL*, showing the Gelsolin-like and headpiece (HP) domains. The coordinates reflect the codon numbers, and the colored bars are the exons. Detailed variant annotation appears in Supplementary Table 19. Panel **a** was created using BioRender.com.

Lead variants at GWAS and MTAG loci map to noncoding sequences of the genome, with only a few exceptions that are missense variants in *BAG3*, *ADPRHL1*, *PROB1* and *RNF207* (Table 1 and Supplementary Tables 8 and 11). Prioritization of potential causal genes in HCM MTAG loci was performed using OpenTargets variant-to-gene (V2G) mapping^13^ (Supplementary Table 12) and FUMA^14^ (Supplementary Table 13). Of all prioritized genes, 26 were selected based on concordance in both OpenTargets (top three genes per locus) and FUMA, as well as LV-specific expression in bulk RNA-seq data (genotype-tissue expression project (GTEx) v.8) and expression in cardiomyocytes using publicly available snRNA-seq data^15^ (Fig. 4a and Supplementary Tables 12 and 13). Of those 26 genes, 14 are in novel loci and include genes involved in cardiomyocyte energetics and metabolism (*RNF207* (ref. ^16^), *MLIP*^17^), myocyte differentiation and transcriptional regulation (*MITF*^18^, *PROX1* (ref. ^19^), *TMEM182* (ref. ^20^)), myofibril assembly (*SVIL*^21^) and calcium handling and contractility (*PDE3A*^22^, *SRL*^23^). To identify further genes associated with HCM, we performed a transcriptome-wide association study (TWAS) using S-MultiXcan^24^ with the MTAG summary statistics and cardiac tissues (LV and AA) from GTEx v.8. TWAS identified 127 genes significantly associated with HCM at *P* < 3.7 × 10^−6^ (Supplementary Table 14), of which 50 were not mapped to MTAG loci using either FUMA or OpenTargets, including *HHATL* (*P* = 1 × 10^−11^)—a gene of uncertain function prioritized based on dominant LV expression and whose depletion in zebrafish may lead to cardiac hypertrophy^25^. Finally, we used OpenTargets to explore association of the 70 lead single nucleotide polymorphisms (SNPs) (or any other SNP in linkage disequilibrium, *r*^2^ > 0.5) with published cardiovascular, metabolic or other traits (Supplementary Table 15). Of the 70 loci associated with HCM, 51 were previously associated at *P* < 5 × 10^−8^ with cardiovascular and/or cardiometabolic traits, including ECG measures, body mass, blood pressure, atrial fibrillation, left ventricular structure/function, atherosclerotic cardiovascular disease and lipids.

GWAS loci often colocalize with genes harboring disease-causing rare variants^26^. To identify novel HCM disease genes, we explored whether rare (MAF < 10^−4^) predicted loss-of-function (LoF) variants in the 26 prioritized genes from significant GWAS/MTAG loci are associated with HCM. We performed case–control burden testing using sequencing data from BioResource of Rare Diseases (BRRD), Genomics England (GEL), UKB and the Oxford Medical Genetics Laboratory (OMGL; only for *SVIL*), followed by a fixed-effects model IVW meta-analysis comprised of up to 2,502 unrelated HCM cases and 486,217 controls (Fig. 4b and Supplementary Table 16a). Rare LoF variants in *ALPK3* and *SVIL* were significantly associated with HCM at the Bonferroni-corrected *P* < 0.0019 (0.05/26). While truncating variants in *ALPK3* have previously been shown to cause HCM and are now included in most clinical testing panels^26–28^, *SVIL* represents a novel HCM gene with a comparable effect size for LoF variants (odds ratio (OR), 10.5,; 95% confidence intervals (CI), 4.3–26.1; *P* = 3.6 × 10^−7^). As exploratory analyses, we also performed exome-wide gene-based burden testing for LoF variants using two MAF filters (< 10^−3^ and <10^−4^) and report the summary statistics in the supplement (Supplementary Tables 17 and 18 and Supplementary Figs. 16–18). Effect estimates for rare *SVIL* LoF variants do not show significant heterogeneity across the four datasets (Supplementary Fig. 19), and the associations remain significant when excluding each dataset one at a time (Supplementary Table 16b). Furthermore, synonymous variant burden testing was performed as a negative control and did not show significant associations (Fig. 4b and Supplementary Table 16c). *SVIL* LoF variants found in eight unrelated cases are listed in Supplementary Table 19 and Fig. 4c. None of the eight unrelated HCM cases that carry a *SVIL* LoF variant carries any other pathogenic or likely pathogenic variant. Family screening provided limited evidence of cosegregation. In one family, variant *SVIL*:p.(Gln255Ter) was carried by two cousins with HCM and, in another family, variant *SVIL*:p.(Arg1616Ter) was carried by two siblings with HCM. *SVIL* encodes supervillin, a large, multidomain actin and myosin binding protein with several muscle and nonmuscle isoforms, of which the muscle isoform has known roles in myofibril assembly and Z-disk attachment^21^. SVIL is highly expressed in cardiac, skeletal and smooth muscle myocytes in the GTEx v.9 snRNA-seq dataset^29^, and *SVIL* morpholino knockdown in zebrafish produces cardiac abnormalities^30^. In humans, LoF *SVIL* variants have been associated with smaller descending aortic diameter^31^, and homozygous LoF *SVIL* variants have been shown to cause a skeletal myopathy with mild cardiac features (left ventricular hypertrophy)^32^. Of interest, common variants in the *SVIL* locus are also associated with dilated cardiomyopathy (DCM)^3^ and, using a Bayesian pairwise analysis approach (GWAS-PW^33^) including the present HCM GWAS meta-analysis and a published DCM GWAS^34^, we show that DCM and HCM share the same causal SNP but with the expected opposite directions of effect (Supplementary Table 20). Taken together, these data support *SVIL* as the likely causal gene in the HCM GWAS locus and identify *SVIL* as a novel disease gene for HCM, in which rare LoF alleles have an effect size similar to that of minor HCM disease genes tested in clinical practice^26–28^.

**Fig. 5:**
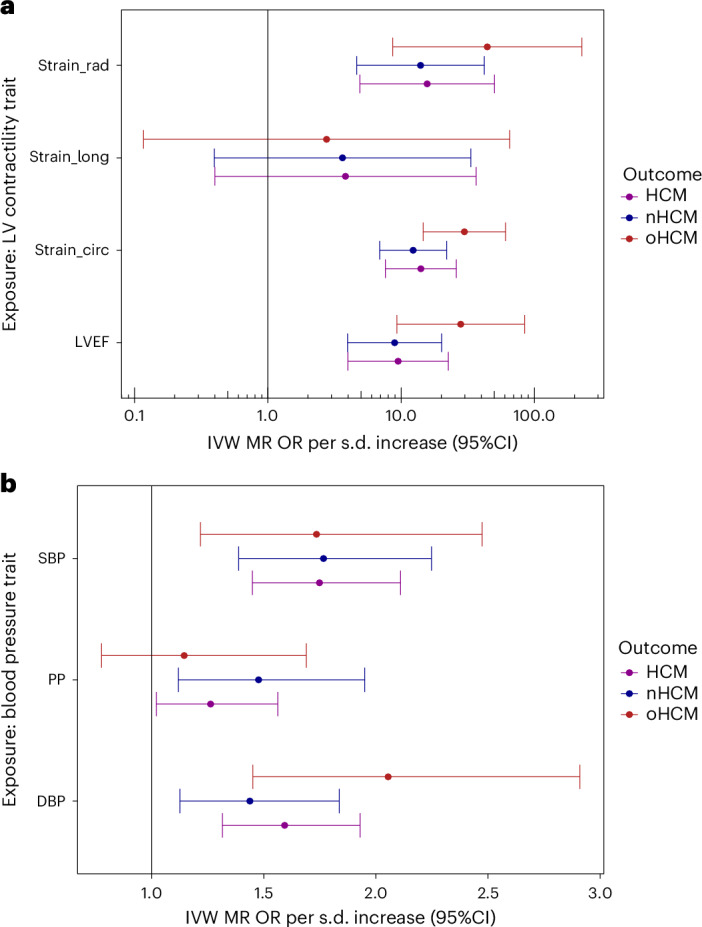
MR analysis of LV contractility and blood pressure on risk of oHCM and nHCM. In both panels, filled circles represent the OR per s.d. increase inferred from the IVW two-sample MR. Error bars represent the 95% CI of the OR. **a**, MR suggests causal association of LV contractility (exposure) with HCM, oHCM and nHCM (outcomes), where increased contractility increases disease risk. Genetic instruments for LV contractility were selected from the present GWAS of LVEF and LV strain in the radial (strain_rad), longitudinal (strain_long) and circumferential (strain_circ) directions in 36,083 participants of the UKB without cardiomyopathy and with available CMR. To facilitate interpretation of effect directions, OR for strain_circ and strain_long reflect those of increased contractility (more negative strain_circ and strain_long values). The outcome HCM GWAS included 5,900 HCM cases versus 68,359 controls. Of those, 964 cases and 27,163 controls were included in the oHCM GWAS and 2,491 cases and 27,109 were included in the nHCM GWAS. Note a logarithmic scale in the *x* axis. **b**, MR suggests causal associations of SBP and DBP with HCM, nHCM and oHCM. Genetic instruments for SBP, DBP and PP (SBP − DBP) were selected from a published GWAS including up to 801,644 people^39^. See Supplementary Table 21 for full MR results.

Rare sarcomeric gene variants that cause HCM have been shown to result in increased contractility, and cardiac myosin inhibitors attenuate the development of sarcomeric HCM in animal models^35^. Previous data from GWAS and Mendelian randomization (MR) also support a causal association of increased LV contractility with HCM, extending beyond rare sarcomeric variants^3^. Pharmacologic modulation of LV contractility using myosin inhibitors has been approved recently in the treatment of HCM associated with LV obstruction (oHCM)^36,37^, but remains of uncertain utility in nHCM where no specific therapy currently exists. To further dissect the specific implication of LV contractility in nHCM and oHCM, we performed two-sample MR, testing the causal association of LV contractility as exposure, with HCM, nHCM and oHCM as outcomes. GWAS of nHCM (2,491 cases) and oHCM (964 cases) were performed (Supplementary Table 2), showing substantially shared genetic basis between nHCM and oHCM (rg = 0.87; s.e. 0.13; *P* = 4 × 10^−11^) (Supplementary Table 8). LV contractility in the general population was assessed with CMR using a volumetric method (LV ejection fraction (LVEF)), and three-dimensional tissue deformation methods (that is, global LV strain in the longitudinal (strain^long^), circumferential (strain^circ^) and radial (strain^rad^) directions). Results from the primary MR inverse variance weighted (IVW) analysis are shown in Fig. 5a, and sensitivity analyses results appear in Supplementary Table 21 and Supplementary Figs. 20 and 21. Although significant heterogeneity in the exposure–outcome effects and potential violations of MR assumptions are possible limitations, MR findings support a causal association between increased LV contractility and increased risk for both nHCM and oHCM, with a substantial risk increase of 12-fold and 29-fold per s.d. increase in the absolute value of strain^circ^, respectively (Fig. 5a). These data suggest that increased contractility is involved in both oHCM and nHCM development, and thus myosin inhibitors currently approved for symptom control in oHCM may also be of clinical benefit in nHCM. Finally, we also performed MR analyses exploring whether increased systolic (SBP) and diastolic (DBP) blood pressure, and pulse pressure (PP = SBP − DBP) are causally associated with nHCM and oHCM. As for LV contractility, the causal association of SBP and DBP with HCM^2^ extended to both oHCM and nHCM subgroups (Fig. 5b, Supplementary Table 21 and Supplementary Fig. 22), suggesting that lowering blood pressure may be a therapeutic target to mitigate disease progression for both nHCM and oHCM.

The large number of new susceptibility loci arising from this work support new inferences regarding disease mechanisms in HCM. With the identification of the role of *SVIL*, we have uncovered further evidence that a subset of genes underlies both monogenic and polygenic forms of the condition. However, this shared genetic architecture does not extend to the core sarcomere genes that cause monogenic HCM; instead, the common variant loci implicate processes outside the myofilament, thereby widening our biological understanding. The shared mechanistic pathways between oHCM and nHCM suggest that the new class of myosin inhibitors may be effective in both settings, whereas the further exploration of newly implicated loci and pathways may in the future yield new treatment targets.

## Methods

### Ethics

The study was approved by the following ethics review boards: Research Ethics and New Technology Development Committee of the Montreal Heart Institute (2011/208), Medical Ethical Committee of Amsterdam University Medical Center (UMC; W20_226 no. 20.260), South Central–Hampshire B Research Ethics Committee (09/H0504/104), Hammersmith and Queen Charlotte’s Research Ethics Committee (09/H0707/69) and the National Research Ethics Service (11/NW/0382, 13/EE/0325, 14/EE/1112, 14/SC/0190, 19/SC/0257, 21/NW/0157). The study of HCM patients from Amsterdam UMC was performed under a waiver—approved by the Medical Ethical Committee of Amsterdam UMC—allowing genotyping and genome-wide association study of people affected by cardiovascular disease. All other study participants provided informed consent.

### GWAS of HCM

The HCM GWAS included cases and controls from seven strata: the Hypertrophic Cardiomyopathy Registry (HCMR), a Canadian HCM cohort, a Netherlands HCM cohort, the Genomics England 100,000 Genome Project (GEL), the Royal Brompton HCM cohort, an Italian HCM cohort and the BioResource for Rare Disease (BRRD) project. Quality control (QC) and association analyses were performed per strata, followed by a meta-analysis. The seven strata are described in the Supplementary Note and in Supplementary Table 1. Cases consisted of unrelated patients diagnosed with HCM in presence of unexplained LV hypertrophy defined as a LV wall thickness (LVWT) > 15 mm, or >13 mm and either presence of family history of HCM or a pathogenic or likely pathogenic genetic variant causing HCM. HCM cases underwent gene panel sequencing as per clinical indications. Variants identified within eight core sarcomere genes (*MYBPC3*, *MYH7*, *TNNI3*, *TNNT2*, *MYL2*, *MYL3*, *ACTC1* and *TPM1*) were assessed centrally at the Oxford laboratory using the American College of Medical Genetics and Genomics (ACMG) guidelines^40^. HCM cases were dichotomized into sarcomere-positive and sarcomere-negative groups using a classification framework previously reported in Neubauer et al.^41^. In addition to the primary all-comer GWAS analyses including all cases with HCM (total of 5,900 cases and 68,359 controls), analyses stratified for sarcomere status in cases and randomly allocated controls were performed, including a total of 1,776 cases versus 29,414 controls in the HCM_SARC+_ analysis and 3,860 cases versus 38,942 controls in the HCM_SARC−_ analysis.

Meta-analyses for the all-comer HCM GWAS was performed on betas and standard errors using GWAMA^42^. We kept variants where meta-analysis came from two or more studies and also had a sample size >5,000. Genomic inflation was estimated from the median *χ*^2^ distribution and using HapMap3 European ancestry LD scores using LD Score Regression^5^. All variants were mapped to Genome Reference Consortium Human Build 37 (GRCh37) extrapolated using the 1000 Genome phase 3 genetic maps. A genome-wide significant locus was assigned where two variants had a meta-analysis *P* < 5 × 10^−8^ and were 0.5 cM distance apart. A similar approach was implemented for the HCM_SARC+_ and HCM_SARC−_ stratified analyses, which comprised five and seven strata, respectively (the GEL and BRRD strata did not include enough HCM_SARC+_ cases). Variants were retained where meta-analysis came from two or more studies and had sample size >5,000 for HCM_SARC−_ and >2,500 for HCM_SARC+_ cases. The final dataset included 9,492,702 (all-comer), 7,614,734 (HCM_SARC+_) and 9,226,079 (HCM_SARC−_) variants after filtering. The results of the all-comer HCM GWAS meta-analysis and stratified analyses are presented in Table 1, Supplementary Figs. 1 and 2 and Supplementary Table 2.

A FDR 1% *P* value cut-off was derived from the all-comer, HCM_SARC+_ and HCM_SARC−_ summary statistics using Simes method (Stata v.10.1), and the corresponding *P* values were 8.5 × 10^–6^, 1.6 × 10^–6^ and 7.8 × 10^–6^, respectively. Using the 1% FDR *P*-value thresholds, we then performed a stepwise model selection to identify 1% FDR independently associated variants using GCTA^4^. The analysis was performed chromosome wise using default window of 10 Mb, 0.9 collinearity and UKB reference panel containing 60,000 unrelated European ancestry participants. The results of this conditional analysis are presented in Supplementary Table 3.

### HCM heritability attributable to common variants

We estimated the heritability of HCM attributable to common genetic variation (*h*^2^_SNP_) in the all-comer HCM, as well as HCM_SARC+_ and HCM_SARC−_ using LDSC^5^ and GREML^6^. For LDSC, HapMap3 SNPs were selected from the summary statistics corresponding to HCM, HCM_SARC+_ and HCM_SARC−_ meta-analyses. The *h*^2^_SNP_ was computed on the liability scale assuming a disease prevalence of 0.002 (ref. ^43^). Since LDSC tends to underestimate *h*^2^_SNP_, we also estimated *h*^2^_SNP_ using GREML, as previously performed^2,3^. We first computed *h*^2^_SNP_ for HCM, HCM_SARC+_ and HCM_SARC−_ using GREML for each of the largest three strata (HCMR, the Canadian HCM cohort and the Netherlands HCM cohort), followed by fixed-effects and random-effects meta-analyses combining all three strata. To exclude the contribution of rare founder HCM causing variants, we excluded the *MYBPC3* locus for the Canadian and Netherlands strata and the *TNNT2* locus for the Canadian stratum^3^. The results of *h*^2^_SNP_ analyses are presented in Supplementary Table 4.

### GWAS of CMR imaging-derived LV traits

#### UKB study population

The UKB is an open-access population cohort resource that has recruited half a million participants in its initial recruitment phase, from 2006 to 2010. At the time of analysis, CMR imaging data were available from 39,559 participants in the imaging substudy. The UKB CMR acquisition protocol has been described previously^44^. In brief, images were acquired according to a basic cardiac imaging protocol using clinical 1.5 T wide bore scanners (MAGNETOM Aera, Syngo Platform VD13A, Siemens Healthcare) in three separate imaging centers. Extensive clinical and questionnaire data and genotypes are available for these participants. Clinical data were obtained at the time of the imaging visit. These included sex (31), age (21003), weight (21002), height (50), SBP (4080), DBP (4079), self-reported noncancer illness code (20002) and ICD-10 codes (41270). The mean age at the time of CMR was 63 ± 8 years (range 45–80) and 46% of participants were male. Cohort anthropometrics, demographics and comorbidities are reported in Supplementary Table 5. Exclusion criteria for the UKB imaging substudy included childhood disease, pregnancy and contraindications to magnetic resonance imaging (MRI) scanning. For the current analysis, we also excluded, by ICD-10 code and/or self-reported diagnoses, anyone with heart failure, cardiomyopathy, a previous myocardial infarction or structural heart disease. After imaging QC and exclusions for comorbidities or genotype QC, we had a maximum cohort size of 36,083 people. The UKB received National Research Ethics Approval (REC reference no. 11/NW/0382). The present study was conducted under terms of UKB access approval no. 18545.

#### LV trait phenotyping

Description of CMR image analysis is detailed in the Supplementary Note and in ref. ^3^. We included ten LV phenotypes for GWAS analyses: end-diastolic volume (LVEDV), end-systolic volume (LVESV), LV ejection fraction (LVEF), mass (LVM), concentricity index (LV concentricity index (LVconc) = LVM/LVEDV), mean wall thickness (meanWT) and maximum wall thickness (maxWT) as well as global peak strain in radial (strain^rad^), longitudinal (strain^long^) and circumferential (strain^circ^) directions. The means and s.d. values of all ten LV phenotypes, overall and stratified by sex, are shown in Supplementary Table 5.

#### LV trait GWAS

A description of genotyping, imputation and QC appears in the Supplementary Note. The GWAS model for LVEF, LVconc, meanWT, maxWT, strain^rad^, strain^long^ and strain^circ^ included age, sex, mean arterial pressure (MAP), body surface area (BSA, derived from the Mosteller formula) and the first eight genotypic principal components as covariates. LVEDV, LVESV and LVM were indexed to BSA for the analysis, as commonly performed in clinical practice. For indexed values (LV end-diastolic volume indexed for BSA (LVEDVi), LV end-systolic volume indexed for BSA (LVESVi) and LV mass indexed for BSA (LVMi), the GWAS model did not include BSA as a covariate, but all other covariates were the same as for nonindexed phenotypes. BOLT-LMM (v.2.3.2)^45^ was used to construct mixed models for association with around 9.5 million directly genotyped and imputed SNPs. A high-quality set of directly genotyped model SNPs was selected to account for random effects in the genetic association analyses. These were selected by MAF (>0.001), and LD-pruned (*r*^2^ < 0.8) to create an optimum SNP set size of around 500,000. The model was then applied to the >9.8 million imputed SNPs passing QC and filtering. Results of the LV traits GWAS are shown in Supplementary Table 6 and Supplementary Figs. 3–12.

#### Locus definition and annotation

Genomic loci associated with all LV traits were annotated jointly. Specifically, summary statistics were combined with a *P* value corresponding to the minimal *P* value (minP) across all ten summary statistics. The minP summary statistics was then used to define loci using FUMA v.1.4.2 (ref. ^14^) using a maximum lead SNP *P* value of 5 × 10^−8^, maximum GWAS *P* value of 0.05 and *r*^2^ threshold for independent significant SNPs of 0.05 (using the European 1000 Genomes Project dataset) and merging LD blocks within 250 kb. Loci were then mapped to genes using positional mapping (<10 kb), expression quantitative trait loci mapping using GTEx v.8 restricted to atrial appendage, left ventricle and skeletal muscle tissues, and chromatin interaction mapping using left and right ventricles. Genes mapped using FUMA were further prioritized by querying the Clinical Genomes Resource (ClinGen)^46^ for genes linked to Mendelian heart disease with moderate, strong or definitive evidence, and using a recent review of overlapping GWAS and Mendelian cardiomyopathy genes^26^. In addition to FUMA locus-to-gene mapping, we also report closest gene and top gene mapped using OpenTargets^13^. Annotated LV trait loci are shown in Supplementary Table 6.

### Genetic correlations between HCM and LV traits

Pairwise genetic correlations for HCM and the ten LV traits were assessed using LDSC v.1.0.1 (ref. ^9^). The analysis was restricted to well-imputed nonambiguous HapMap3 SNPs, excluding SNPs with MAF < 0.01 and those with low sample size, using default parameters. We then assessed genetic correlations for each of the 55 pairs (HCM and ten LV traits) using precomputed LD scores from the European 1000 Genomes Project dataset. We did not constrain the single-trait and cross-trait LD score regression intercepts. The results of the genetic correlation analyses are shown in Fig. 2 and Supplementary Table 7.

### Multitrait analysis of GWAS

We performed multitrait analysis of GWAS summary statistics using MTAG (v.1.0.8)^7^ to increase power for discovery of genetic loci associated with HCM. MTAG jointly analyzes several sets of GWAS summary statistics of genetically correlated traits to enhance statistical power. Due to high computation needs to calculate the maxFDR with MTAG, we limited the number of GWAS summary statistics to four (HCM plus three LV traits). The three LV traits to include were selected as follows. First, we performed hierarchical clustering of the ten LV traits using the absolute value of the pairwise genetic correlations, Euclidean distance and the complete method, predefining the number of clusters to three. This resulted in clustering of LV traits into an LV contractility cluster (LVEF, strain^rad^, strain^long^ and strain^circ^), an LV volume cluster (LVEDVi, LVESVi) and an LV mass cluster (LVMi, LVconc, meanWT, maxWT) (Fig. 2). We then selected the trait with the highest genetic correlation with HCM from each cluster (strain^circ^, LVESVi and LVconc) to include in MTAG together with HCM. Only SNPs included in all meta-analyses (that is, HCM and LV traits) were used in MTAG. The coded/noncoded alleles were aligned for all four studies before MTAG, and multi-allelic SNPs were removed. All summary statistics refer to the positive strand of GRCh37 and, as such, ambiguous/palindromic SNPs (having alleles A/T or C/G) were not excluded. Regression coefficients (beta) and their s.e. were used as inputs for MTAG. The maxFDR was calculated as suggested by the MTAG developers^7^. MaxFDR calculates the type I error in the analyzed dataset for the worst-case scenario. We estimated the gain in statistical power by the increment in the *N*_eff_. The *N*_eff_ for the HCM GWAS was calculated using the following formula^7,47^:

$${N}_{{\rm{eff}}({\rm{GWAS}})}=\frac{4}{{{N_{\rm{cases}}}}^{-1}+{{N_{\rm{controls}}}}^{-1}}$$

The *N*_eff_ for the HCM MTAG was computed by means of the fold-increase in mean *χ*^2^, using the following formula^7^, implemented in MTAG, where the MTAG *N*_eff_ corresponds to the approximate sample size needed to achieve the same mean *χ*^2^ value in a standard GWAS:

$${N}_{{\rm{eff}}({\rm{MTAG}})}={N}_{{\rm{eff}}({\rm{GWAS}})}\times \left({\chi }_{{\rm{MTAG}},{\rm{mean}}}^{2}-1/{\chi }_{{\rm{GWAS}},{\rm{mean}}}^{2}-1\right)$$

To explore whether HCM effects estimates derived from MTAG are accurate, we compared the regression coefficients derived from MTAG with those derived from GWAS. This was performed for all variants included in MTAG and GWAS, and for a subset of variants reaching nominal significance (*P* < 0.001) in either GWAS and/or MTAG (Supplementary Fig. 13). The results of HCM MTAG are presented in Fig. 3, Supplementary Table 8 and Supplementary Fig. 14.

### Genome-wide annotation and gene set enrichment analyses

Genome-wide analyses following MTAG were performed using MAGMA v.1.08, as implemented in FUMA^14^, including gene set and tissue expression analyses. We used Gene Ontology gene sets from the Molecular Signatures Database (MsigDB, v.6.2) for the gene set analysis and GTEx v.8 for the tissue specificity analysis. The results of MAGMA analyses are shown in Supplementary Table 9 (gene set analyses) and Supplementary Table 10 (tissue specificity analyses).

### Cardiac cell type heritability enrichment analysis

Gene programs derived from snRNA-seq were used to investigate heritability enrichment in cardiac cell types and states using the sc-linker framework^12^. This approach uses snRNA-seq data to generate gene programs that characterize individual cell types and states. These programs are then linked to genomic regions and the SNPs that regulate them by incorporating Roadmap Enhancer-Gene Linking^48,49^ and Activity-by-Contact models^50,51^. Finally, the disease informativeness of resulting SNP annotations was tested using stratified LDSC (S-LDSC)^52^ conditional on broad sets of annotations from the baseline-LD model^53,54^. Cell type and state-specific gene programs were generated from snRNA-seq data of ventricular tissue from 12 control participants, with cell type and state annotations made as part of a larger study of ~880,000 nuclei (samples from 61 DCM and 12 control participants^11^). Cell states that may not represent true biological states (for example, technical doublets) were excluded from analysis. Results of sc-linker cardiac cell type heritability enrichment analysis are shown in Supplementary Fig. 15.

### Locus-to-gene annotation

A genome-wide significant HCM MTAG locus was assigned where two variants had a MTAG *P* < 5 × 10^–8^ and were 0.5 cM distance apart, as performed for the HCM GWAS. Prioritization of potential causal genes in HCM MTAG loci was performed using OpenTargets V2G mapping^13^ and FUMA^14^. The lead SNP at each independent locus was used as input for OpenTargets V2G using the release of 12 October 2022. Locus-to-gene mapping with FUMA v.1.3.7 was performed based on (1) position (within 100 kb), (2) expression quantitative trait loci associations in disease-relevant tissues (GTEx v.8 left ventricle, atrial appendage and skeletal muscle) and (3) chromatin interactions in cardiac tissue (left ventricle and right ventricle, FDR < 10^−6^).

We further annotated genes mapped using OpenTargets and/or FUMA with their implication in Mendelian cardiomyopathy. Specifically, we queried the Clinical Genome Resource (ClinGen^28,46^) for genes associated with any cardiomyopathy phenotype with a level of evidence of moderate, strong or definitive and included genes with robust recent data supporting an association with Mendelian cardiomyopathy^26^.

We also prioritized genes based on RNA expression data from bulk tissue RNA-seq data in the GTEx^38^ v.8 dataset accessible at the GTEx Portal and snRNA-seq data from Chaffin et al.^15^ accessible through the Broad Institute Single Cell Portal (https://singlecell.broadinstitute.org/single_cell). Using the GTEx v.8 data, we assessed specificity of LV expression by computing the ratio of median LV transcripts per million (TPM) to the median TPM in other tissues excluding atrial appendage and skeletal muscle and averaging tissue within types (for example, all arterial tissues, all brain tissues and so on). High and Mid LV expression specificity were empirically defined as >10-fold and >1.5-fold LV to other tissues median TPM ratios, respectively. Using snRNA-seq data from Chaffin et al.^15^, we report the expression in the cardiomyocyte_1 cell type using scaled mean expression (relative to each gene’s expression across all cell types) and percentage of cells expressing. High and Mid expression in cardiomyocytes were empirically defined as percentage expressing cells ≥80% and 40–80%, respectively. Prioritized genes were defined as genes mapped using both OpenTargets (top three genes) and FUMA, and had either (1) High LV-specific expression, (2) High cardiomyocyte expression or (3) both Mid LV-specific expression and Mid cardiomyocyte expression.

Lead variants in MTAG and GWAS loci were also annotated using the Ensembl Variant Effect Predictor (VEP) and lookup of lead variants and variants in LD (*r*^*2*^ > 0.5) in other GWAS was performed using OpenTargets genetics.

Gene mapping and variant annotation data are shown in Supplementary Table 11 (VEP annotation), Supplementary Table 12 (OpenTargets genes), Supplementary Table 13 (FUMA genes) and Supplementary Table 15 (lookup in other GWAS). Prioritized genes are illustrated in Fig. 4a.

### Transcriptome-wide association study

We used MetaXcan to test the association between genetically predicted gene expression and HCM using summary results from MTAG analysis^24,55^. Biologically informed MASHR-based prediction models of gene expression for LV and AA tissue from GTEx v.8 (ref. ^56^) were analyzed individually with S-PrediXcan^55^, and then analyzed together using S-MultiXcan^24^. GWAS MTAG summary statistics were harmonized and imputed to match GTEx v.8 reference variants present in the prediction model. To account for multiple testing, TWAS significance was adjusted for the total number of genes present in S-MultiXcan analysis (13,558 genes, *P* = 3.7 × 10^–6^). TWAS results are shown in Supplementary Table 14.

### Association of rare LoF variants in prioritized genes with HCM

We assessed the association of rare LoF variants in each of 26 prioritized genes (Fig. 4a) with HCM using burden analysis in three primary cohorts (BRRD, GEL and UKB) followed by fixed-effect meta-analysis. For BRRD, HCM cases were probands within the bio-resource project HCM. Controls were all remaining participants within the BRRD projects excluding those also recruited into the GEL and GEL2 projects (Genomics England pilot data). For GEL, HCM cases were probands referred into GEL with a primary clinical diagnosis of HCM. Controls were probands without any primary or secondary cardiovascular disease or myopathy. For UKB, HCM cases were identified from self-reported questionnaires at study recruitment, ICD-10 codes from clinical admission data and death registries, and CMR imaging for the subset of the cohort who underwent cardiac MRI testing (LV maximum wall thickness >15 mm). All participants with aortic stenosis were excluded from UKB cases. Sequencing data were available for only *SVIL* in the Oxford Medical Genetics Laboratory (OMGL), where cases were clinically diagnosed with HCM and referred for diagnostic panel testing. The control group for the OMGL analysis consisted of 5,000 white British ancestry and unrelated control participants selected randomly from the UKB; these participants had normal LV volume and function and no clinical diagnosis of any cardiomyopathy. The remaining UKB samples were used as controls for UKB burden analysis. Genetic variants were identified using next-generation sequencing (whole-genome sequencing for BRRD, GEL, panel/exome sequencing for OMGL cases and UKB) and annotated using VEP and LOFTEE plugin^57^. LoF variants were defined as those with the following VEP terms: stop lost, stop gained, splice donor variant, splice acceptor variant and frameshift variant. Only variants with a MAF < 10^−4^ in the non-Finnish European (NFE) ancestral group of gnomAD v.2.1.1 (ref. ^58^) were selected. LoF variants present in the Matched Annotation from NCBI and EMBL-EBI (MANE)/canonical transcript or next best transcript were retained for the analysis. The proportion of cases and controls with LoF variants were compared using the Fisher Exact test for each of the BRRD, GEL and OMGL datasets. UKB LoF burden test was performed using their REGENIE workflow. We included high-quality sequenced variants where >90% samples had a sequencing depth >10, and tested genes with a minor allele count of ≥10. Firth correction was used to account for inflation resulting from case–control imbalance in the UKB. As a negative control, we also performed association testing of rare (MAF < 10^–4^) synonymous variants for each of the 26 prioritized genes using an identical methodology. Meta-analysis of burden test results was performed using the IVW method including studies with no zero counts and estimating standard error using sample counts^59^. The results of LoF and synonymous variant association with HCM are shown in Supplementary Table 16 and Fig. 4b. Further results for *SVIL* LoF variant analyses are shown in Fig. 4c, Supplementary Fig. 19 and Supplementary Table 19.

An exploratory exome-wide gene-based burden testing for LoF variants was also performed, using two MAF thresholds (<10^−4^ and <10^−3^). For UKB, this exploratory exome-wide analysis was performed as for the targeted analysis described above. For GEL and BRRD, this analysis was performed on the corresponding GEL and BRRD servers, using pre-annotated (VEP) files and filtering for LoF variants with gnomAD NFE AF < 10^−3^. For BRRD, variants were lifted to human genome build GRCh38 and LOFTEE was used to select high confidence LoF variants. For OMGL, only rare *SVIL* variants were available for analysis. Gene-based rare variant burden analyses followed by meta-analysis were performed as mentioned in the preceding paragraph. A sample size weighted meta-analysis was also performed, using the *N*_eff_ formula shown above. The results of these exploratory exome-wide gene-based analyses are shown in Supplementary Tables 17 and 18 (full summary statistics) and Supplementary Figs. 16–18 (quantile–quantile and Manhattan plots).

### Locus colocalization in DCM and HCM

We explored colocalization of HCM and DCM loci using GWAS-PW^33^. The genome was split into 1,754 approximately independent regions and the all-comer HCM meta-analysis results were analyzed with those of a publicly available DCM GWAS^34^ using a Bayesian approach. GWAS-PW fits each locus into one of the four models where model 1 is association in only the first trait, model 2 is association in only the second trait, model 3 when the two traits colocalize, and model 4 when the genetic signals are independent in the two traits. We considered a locus to show colocalization when either trait harbors a genetic signal with *P* < 1 × 10^–5^ and the GWAS-PW analysis demonstrates a posterior probability of association for model 3 (PPA3) >0.8. Results of GWAS-PW are presented in Supplementary Table 20.

### Two-sample MR

We assessed whether increased contractility and blood pressure are causally linked to increased risk of HCM globally and its obstructive (oHCM) and non-obstructive (nHCM) forms using two-sample MR. LV contractility and blood pressure parameters were used as exposure variables, and HCM, oHCM and nHCM as outcomes. Analyses were performed using the TwoSampleMR (MRbase) package^60^ (v.0.5.6) in R (v.4.2.0). Four exposure variables corresponding to measures of LV contractility were used separately: LVEF as a volumetric marker of contractility, and global strain (strain^circ^, strain^rad^ and strain^long^) as contractility markers based on myocardial tissue deformation. Instrument SNPs for contractility were selected based on the LV trait GWAS presented here using a *P* value threshold of <5 × 10^−8^. Only independent SNPs (using *r*^2^ < 0.01 in the European 1000 Genomes population) were included. Instrument SNPs for the blood pressure analysis were selected with a similar approach using a published blood pressure GWAS^39^. The outcome summary statistics were those of the single-trait HCM case–control meta-analysis (5,900 cases and 68,359 controls). We also performed a GWAS meta-analysis including data from HCMR and the Canadian HCM cohort (Supplementary Table 1) for nHCM (2,491 cases and 27,109 controls) and oHCM (964 cases and 27,163 controls) to use as outcomes. For these stratified analyses, oHCM was defined as HCM in presence of a LV outflow tract gradient ≥30 mmHg at rest or during Valsalva/exercise at any timepoint. All other HCM cases were considered nHCM. Loci reaching *P* < 5 × 10^−8^ in oHCM and nHCM are shown in Supplementary Table 2 and lookup of all-comer HCM MTAG loci in oHCM and nHCM are shown in Supplementary Table 8.

Insertions/deletions and palindromic SNPs with intermediate allele frequencies (MAF > 0.42) were excluded, and other SNPs in the same locus were included only if *P* < 5 × 10^−8^. An inverse variance weighted MR model was used as a primary analysis. We used three additional methods as sensitivity analyses: weighted median, weighted mode and MR Egger. Cochran’s *Q* statistics were calculated to investigate heterogeneity between SNP causal effects using IVW. Evidence of directional pleiotropy was also assessed using the MR Egger intercept. Mean *F*-statistics were calculated to assess the strength of the genetic instruments used. Leave-one-out analyses were also performed to ensure the SNP causal effects are not driven by a particular SNP. To further explore the impact of pleiotropy in the contractility/HCM MR analysis and to evaluate the consequence of excluding outlier SNPs, we used the MR pleiotropy residual sum and outlier (MR-PRESSO) analysis^61^. MR-PRESSO consists of three steps: testing for horizontal pleiotropy (global test), correcting for horizontal pleiotropy using outlier removal (outlier test) and evaluating differences in the causal estimate before and after outlier removal (distortion test). The summary results of MR analyses and sensitivity analyses are shown in Fig. 5 and Supplementary Table 21, with effect plots shown in Supplementary Fig. 20 (contractility) and Supplementary Fig. 22 (blood pressure), and leave-one-out analyses for the contractility MR in Supplementary Fig. 21. The MR effects are shown per unit change (percentage for contractility; mmHg for blood pressure) in Supplementary Table 21 and Supplementary Figs. 20–22, and per s.d. change in Fig. 5. OR per s.d. increase are calculated as ${\rm{OR}}={e}^{{\beta }_{{\rm{MR}}}\times {\rm{s.d.}}}$; s.d. values are reported in Supplementary Table 21 and correspond to those in the current UKB CMR dataset (for contractility) and those reported by Evangelou et al.^39^ in the UKB (for blood pressure).

### Reporting summary

Further information on research design is available in the Nature Portfolio Reporting Summary linked to this article.

## Online content

Any methods, additional references, Nature Portfolio reporting summaries, source data, extended data, supplementary information, acknowledgements, peer review information; details of author contributions and competing interests; and statements of data and code availability are available at 10.1038/s41588-025-02087-4.

## Supplementary information

Supplementary Information Supplementary Figs. 1–22 and Note.

Reporting Summary

Peer Review File

Supplementary Tables Supplementary Tables 1–21.

## Data Availability

Data from the Genome Aggregation Database (gnomAD, v.2.1.1) are available at https://gnomad.broadinstitute.org. Data from the UKB can be requested from the UKB Access Management System (https://bbams.ndph.ox.ac.uk). Data from the GTEx consortium are available at the GTEx Portal (https://gtexportal.org). Published snRNA-seq data are available at the Broad Single Cell Portal (https://singlecell.broadinstitute.org/) and at the Cellxgene tool website (https://cellxgene.cziscience.com/collections/e75342a8-0f3b-4ec5-8ee1-245a23e0f7cb/private). The Genome assembly GRCh37 can be accessed using https://www.ncbi.nlm.nih.gov/datasets/genome/GCF_000001405.13/. Individual-level data sharing is subject to restrictions imposed by patient consent and local ethics review boards. Full GWAS summary statistics of HCM, HCM_SARC−_, HCM_SARC+_, MTAG and ten LV traits are available on the GWAS catalog (accession IDs GCST90435254–GCST90435267) and can be accessed interactively at www.well.ox.ac.uk/hcm.

## Code availability

The analyses reported in this manuscript rely on previously published software, as detailed in Methods and in the Reporting Summary.

## References

[1] Watkins, H. Time to think differently about sarcomere-negative hypertrophic cardiomyopathy. *Circulation* **143**, 2415–2417 (2021). 34152793 10.1161/CIRCULATIONAHA.121.053527

[2] Harper, A. R. et al. Common genetic variants and modifiable risk factors underpin hypertrophic cardiomyopathy susceptibility and expressivity. *Nat. Genet.* **53**, 135–142 (2021). 33495597 10.1038/s41588-020-00764-0 PMC8240954

[3] Tadros, R. et al. Shared genetic pathways contribute to risk of hypertrophic and dilated cardiomyopathies with opposite directions of effect. *Nat. Genet.* **53**, 128–134 (2021). 33495596 10.1038/s41588-020-00762-2 PMC7611259

[4] Yang, J. et al. Conditional and joint multiple-SNP analysis of GWAS summary statistics identifies additional variants influencing complex traits. *Nat. Genet.* **44**, 369–375 (2012). 22426310 10.1038/ng.2213 PMC3593158

[5] Bulik-Sullivan, B. K. et al. LD Score regression distinguishes confounding from polygenicity in genome-wide association studies. *Nat. Genet.* **47**, 291–295 (2015). 25642630 10.1038/ng.3211 PMC4495769

[6] Lee, S. H., Wray, N. R., Goddard, M. E. & Visscher, P. M. Estimating missing heritability for disease from genome-wide association studies. *Am. J. Hum. Genet.* **88**, 294–305 (2011). 21376301 10.1016/j.ajhg.2011.02.002 PMC3059431

[7] Turley, P. et al. Multi-trait analysis of genome-wide association summary statistics using MTAG. *Nat. Genet.* **50**, 229–237 (2018). 29292387 10.1038/s41588-017-0009-4 PMC5805593

[8] Bai, W. et al. A population-based phenome-wide association study of cardiac and aortic structure and function. *Nat. Med.* **26**, 1654–1662 (2020). 32839619 10.1038/s41591-020-1009-y PMC7613250

[9] Bulik-Sullivan, B. et al. An atlas of genetic correlations across human diseases and traits. *Nat. Genet.* **47**, 1236–1241 (2015). 26414676 10.1038/ng.3406 PMC4797329

[10] de Leeuw, C. A., Mooij, J. M., Heskes, T. & Posthuma, D. MAGMA: generalized gene-set analysis of GWAS data. *PLoS Comput. Biol.* **11**, e1004219 (2015). 25885710 10.1371/journal.pcbi.1004219 PMC4401657

[11] Reichart, D. et al. Pathogenic variants damage cell composition and single cell transcription in cardiomyopathies. *Science* **377**, eabo1984 (2022). 35926050 10.1126/science.abo1984 PMC9528698

[12] Jagadeesh, K. A. et al. Identifying disease-critical cell types and cellular processes by integrating single-cell RNA-sequencing and human genetics. *Nat. Genet.* **54**, 1479–1492 (2022). 36175791 10.1038/s41588-022-01187-9 PMC9910198

[13] Mountjoy, E. et al. An open approach to systematically prioritize causal variants and genes at all published human GWAS trait-associated loci. *Nat. Genet.* **53**, 1527–1533 (2021). 34711957 10.1038/s41588-021-00945-5 PMC7611956

[14] Watanabe, K., Taskesen, E., van Bochoven, A. & Posthuma, D. Functional mapping and annotation of genetic associations with FUMA. *Nat. Commun.* **8**, 1826 (2017). 29184056 10.1038/s41467-017-01261-5 PMC5705698

[15] Chaffin, M. et al. Single-nucleus profiling of human dilated and hypertrophic cardiomyopathy. *Nature* **608**, 174–180 (2022). 35732739 10.1038/s41586-022-04817-8 PMC12591363

[16] Mizushima, W. et al. The novel heart-specific RING finger protein 207 is involved in energy metabolism in cardiomyocytes. *J. Mol. Cell. Cardiol.* **100**, 43–53 (2016). 27677939 10.1016/j.yjmcc.2016.09.013

[17] Cattin, M. E. et al. Deletion of MLIP (muscle-enriched A-type lamin-interacting protein) leads to cardiac hyperactivation of Akt/mammalian target of rapamycin (mTOR) and impaired cardiac adaptation. *J. Biol. Chem.* **290**, 26699–26714 (2015). 26359501 10.1074/jbc.M115.678433 PMC4646324

[18] Tshori, S. et al. Transcription factor MITF regulates cardiac growth and hypertrophy. *J. Clin. Invest.* **116**, 2673–2681 (2006). 16998588 10.1172/JCI27643 PMC1570375

[19] Risebro, C. A. et al. Prox1 maintains muscle structure and growth in the developing heart. *Development* **136**, 495–505 (2009). 19091769 10.1242/dev.030007 PMC2655234

[20] Luo, W. et al. TMEM182 interacts with integrin beta 1 and regulates myoblast differentiation and muscle regeneration. *J. Cachexia Sarcopenia Muscle* **12**, 1704–1723 (2021). 34427057 10.1002/jcsm.12767 PMC8718073

[21] Lee, M. A. et al. Archvillin anchors in the Z-line of skeletal muscle via the nebulin C-terminus. *Biochem. Biophys. Res. Commun.* **374**, 320–324 (2008). 18639526 10.1016/j.bbrc.2008.07.036

[22] Beca, S. et al. Phosphodiesterase type 3A regulates basal myocardial contractility through interacting with sarcoplasmic reticulum calcium ATPase type 2a signaling complexes in mouse heart. *Circ. Res.* **112**, 289–297 (2013). 23168336 10.1161/CIRCRESAHA.111.300003 PMC3579621

[23] Yoshida, M. et al. Impaired Ca2+ store functions in skeletal and cardiac muscle cells from sarcalumenin-deficient mice. *J. Biol. Chem.* **280**, 3500–3506 (2005). 15569689 10.1074/jbc.M406618200

[24] Barbeira, A. N. et al. Integrating predicted transcriptome from multiple tissues improves association detection. *PLoS Genet.* **15**, e1007889 (2019). 30668570 10.1371/journal.pgen.1007889 PMC6358100

[25] Shi, X. et al. Zebrafish *hhatla* is involved in cardiac hypertrophy. *J. Cell. Physiol.* **236**, 3700–3709 (2021). 33052609 10.1002/jcp.30106

[26] Walsh, R., Offerhaus, J. A., Tadros, R. & Bezzina, C. R. Minor hypertrophic cardiomyopathy genes, major insights into the genetics of cardiomyopathies. *Nat. Rev. Cardiol.* **19**, 151–167 (2022). 34526680 10.1038/s41569-021-00608-2

[27] Biddinger, K. J. et al. Rare and common genetic variation underlying the risk of hypertrophic cardiomyopathy in a national biobank. *JAMA Cardiol.* **7**, 715–722 (2022). 35583889 10.1001/jamacardio.2022.1061 PMC9118016

[28] Ingles, J. et al. Evaluating the clinical validity of hypertrophic cardiomyopathy genes. *Circ. Genom. Precis. Med.* **12**, e002460 (2019). 30681346 10.1161/CIRCGEN.119.002460 PMC6410971

[29] Eraslan, G. et al. Single-nucleus cross-tissue molecular reference maps toward understanding disease gene function. *Science* **376**, eabl4290 (2022). 35549429 10.1126/science.abl4290 PMC9383269

[30] Deo, R. C. et al. Prioritizing causal disease genes using unbiased genomic features. *Genome Biol.* **15**, 534 (2014). 25633252 10.1186/s13059-014-0534-8 PMC4279789

[31] Pirruccello, J. P. et al. Deep learning enables genetic analysis of the human thoracic aorta. *Nat. Genet.* **54**, 40–51 (2022). 34837083 10.1038/s41588-021-00962-4 PMC8758523

[32] Hedberg-Oldfors, C. et al. Loss of supervillin causes myopathy with myofibrillar disorganization and autophagic vacuoles. *Brain* **143**, 2406–2420 (2020). 32779703 10.1093/brain/awaa206 PMC7447519

[33] Pickrell, J. K. et al. Detection and interpretation of shared genetic influences on 42 human traits. *Nat. Genet.* **48**, 709–717 (2016). 27182965 10.1038/ng.3570 PMC5207801

[34] Aragam, K. G. et al. Phenotypic refinement of heart failure in a national biobank facilitates genetic discovery. *Circulation* **139**, 489–501 (2019). 30586722 10.1161/CIRCULATIONAHA.118.035774 PMC6511334

[35] Green, E. M. et al. A small-molecule inhibitor of sarcomere contractility suppresses hypertrophic cardiomyopathy in mice. *Science* **351**, 617–621 (2016). 26912705 10.1126/science.aad3456 PMC4784435

[36] Olivotto, I. et al. Mavacamten for treatment of symptomatic obstructive hypertrophic cardiomyopathy (EXPLORER-HCM): a randomised, double-blind, placebo-controlled, phase 3 trial. *Lancet* **396**, 759–769 (2020). 32871100 10.1016/S0140-6736(20)31792-X

[37] Desai, M. Y. et al. Myosin inhibition in patients with obstructive hypertrophic cardiomyopathy referred for septal reduction therapy. *J. Am. Coll. Cardiol.* **80**, 95–108 (2022). 35798455 10.1016/j.jacc.2022.04.048

[38] GTEx Consortium. The Genotype-Tissue Expression (GTEx) project. *Nat. Genet.* **45**, 580–585 (2013). 23715323 10.1038/ng.2653 PMC4010069

[39] Evangelou, E. et al. Genetic analysis of over 1 million people identifies 535 new loci associated with blood pressure traits. *Nat. Genet.* **50**, 1412–1425 (2018). 30224653 10.1038/s41588-018-0205-x PMC6284793

[40] Richards, S. et al. Standards and guidelines for the interpretation of sequence variants: a joint consensus recommendation of the American College of Medical Genetics and Genomics and the Association for Molecular Pathology. *Genet. Med.* **17**, 405–424 (2015). 25741868 10.1038/gim.2015.30 PMC4544753

[41] Neubauer, S. et al. Distinct subgroups in hypertrophic cardiomyopathy in the NHLBI HCM Registry. *J. Am. Coll. Cardiol.* **74**, 2333–2345 (2019). 31699273 10.1016/j.jacc.2019.08.1057 PMC6905038

[42] Magi, R. & Morris, A. P. GWAMA: software for genome-wide association meta-analysis. *BMC Bioinf.* **11**, 288 (2010). 10.1186/1471-2105-11-288 PMC2893603 20509871

[43] Semsarian, C., Ingles, J., Maron, M. S. & Maron, B. J. New perspectives on the prevalence of hypertrophic cardiomyopathy. *J. Am. Coll. Cardiol.* **65**, 1249–1254 (2015). 25814232 10.1016/j.jacc.2015.01.019

[44] Petersen, S. E. et al. UK Biobank’s cardiovascular magnetic resonance protocol. *J. Cardiovasc. Magn. Reson.* **18**, 8 (2016). 26830817 10.1186/s12968-016-0227-4 PMC4736703

[45] Loh, P. R. et al. Efficient Bayesian mixed-model analysis increases association power in large cohorts. *Nat. Genet.* **47**, 284–290 (2015). 25642633 10.1038/ng.3190 PMC4342297

[46] Rehm, H. L. et al. ClinGen—the Clinical Genome Resource. *N. Engl. J. Med.* **372**, 2235–2242 (2015). 26014595 10.1056/NEJMsr1406261 PMC4474187

[47] Willer, C. J., Li, Y. & Abecasis, G. R. METAL: fast and efficient meta-analysis of genomewide association scans. *Bioinformatics* **26**, 2190–2191 (2010). 20616382 10.1093/bioinformatics/btq340 PMC2922887

[48] Ernst, J. et al. Mapping and analysis of chromatin state dynamics in nine human cell types. *Nature* **473**, 43–49 (2011). 21441907 10.1038/nature09906 PMC3088773

[49] Roadmap Epigenomics Consortium et al. Integrative analysis of 111 reference human epigenomes. *Nature* **518**, 317–330 (2015). 25693563 10.1038/nature14248 PMC4530010

[50] Fulco, C. P. et al. Activity-by-contact model of enhancer-promoter regulation from thousands of CRISPR perturbations. *Nat. Genet.* **51**, 1664–1669 (2019). 31784727 10.1038/s41588-019-0538-0 PMC6886585

[51] Nasser, J. et al. Genome-wide enhancer maps link risk variants to disease genes. *Nature* **593**, 238–243 (2021). 33828297 10.1038/s41586-021-03446-x PMC9153265

[52] Finucane, H. K. et al. Partitioning heritability by functional annotation using genome-wide association summary statistics. *Nat. Genet.* **47**, 1228–1235 (2015). 26414678 10.1038/ng.3404 PMC4626285

[53] Gazal, S. et al. Linkage disequilibrium-dependent architecture of human complex traits shows action of negative selection. *Nat. Genet.* **49**, 1421–1427 (2017). 28892061 10.1038/ng.3954 PMC6133304

[54] Gazal, S., Marquez-Luna, C., Finucane, H. K. & Price, A. L. Reconciling S-LDSC and LDAK functional enrichment estimates. *Nat. Genet.* **51**, 1202–1204 (2019). 31285579 10.1038/s41588-019-0464-1 PMC7006477

[55] Barbeira, A. N. et al. Exploring the phenotypic consequences of tissue specific gene expression variation inferred from GWAS summary statistics. *Nat. Commun.* **9**, 1825 (2018). 29739930 10.1038/s41467-018-03621-1 PMC5940825

[56] Barbeira, A. N. et al. Exploiting the GTEx resources to decipher the mechanisms at GWAS loci. *Genome Biol* **22**, 49 (2021). 33499903 10.1186/s13059-020-02252-4 PMC7836161

[57] McLaren, W. et al. Deriving the consequences of genomic variants with the Ensembl API and SNP Effect Predictor. *Bioinformatics* **26**, 2069–2070 (2010). 20562413 10.1093/bioinformatics/btq330 PMC2916720

[58] Karczewski, K. J. et al. The mutational constraint spectrum quantified from variation in 141,456 humans. *Nature* **581**, 434–443 (2020). 32461654 10.1038/s41586-020-2308-7 PMC7334197

[59] Khan, S. in *Meta-Analysis: Methods for Health and Experimental Studies*, 87–118 (Springer, 2020).

[60] Hemani, G. et al. The MR-Base platform supports systematic causal inference across the human phenome. *eLife* **7**, e34408 (2018). 29846171 10.7554/eLife.34408 PMC5976434

[61] Verbanck, M., Chen, C. Y., Neale, B. & Do, R. Detection of widespread horizontal pleiotropy in causal relationships inferred from Mendelian randomization between complex traits and diseases. *Nat. Genet.* **50**, 693–698 (2018). 29686387 10.1038/s41588-018-0099-7 PMC6083837

