# Supplementary material for: Large-scale genome-wide association analyses identify novel genetic loci and mechanisms in hypertrophic cardiomyopathy
Source: Nat Genet. 2025 Feb 18;57(3):530–8. doi: 10.1038/s41588-025-02087-4 (PMC11906354; doi:10.1038/s41588-025-02087-4)
Supplement: Supplementary file 2 — Reporting Summary [file 41588_2025_2087_MOESM2_ESM.pdf]

nature portfolio

Corresponding author(s): Rafik Tadros, James Ware, Connie Bezzina, and Hugh Watkins

Last updated by author(s): Sep 13, 2024

Reporting Summary

Nature Portfolio wishes to improve the reproducibility of the work that we publish. This form provides structure for consistency and transparency in reporting. For further information on Nature Portfolio policies, see our Editorial Policies and the Editorial Policy Checklist.

Statistics

For all statistical analyses, confirm that the following items are present in the figure legend, table legend, main text, or Methods section.

n/a	Confirmed	
☐	☒	The exact sample size (*n*) for each experimental group/condition, given as a discrete number and unit of measurement
☐	☒	A statement on whether measurements were taken from distinct samples or whether the same sample was measured repeatedly
☐	☒	The statistical test(s) used AND whether they are one- or two-sided<br>*Only common tests should be described solely by name; describe more complex techniques in the Methods section.*
☐	☒	A description of all covariates tested
☐	☒	A description of any assumptions or corrections, such as tests of normality and adjustment for multiple comparisons
☐	☒	A full description of the statistical parameters including central tendency (e.g. means) or other basic estimates (e.g. regression coefficient) AND variation (e.g. standard deviation) or associated estimates of uncertainty (e.g. confidence intervals)
☐	☒	For null hypothesis testing, the test statistic (e.g. *F*, *t*, *r*) with confidence intervals, effect sizes, degrees of freedom and *P* value noted<br>*Give P values as exact values whenever suitable.*
☒	☐	For Bayesian analysis, information on the choice of priors and Markov chain Monte Carlo settings
☒	☐	For hierarchical and complex designs, identification of the appropriate level for tests and full reporting of outcomes
☐	☒	Estimates of effect sizes (e.g. Cohen's *d*, Pearson's *r*), indicating how they were calculated

Our web collection on statistics for biologists contains articles on many of the points above.

Software and code

Policy information about availability of computer code

Data collection	No software used for data collection.
Data analysis	The following publicly available software were used in data analysis:<br>PLINK 1.9 (https://www.cog-genomics.org/plink)<br>SNPTEST 2.5 (https://www.well.ox.ac.uk/~gav/snptest)<br>SAIGE v0.29.4.2 (https://github.com/weizhouUMICH/SAIGE)<br>SHAPEIT v2.r790 (https://mathgen.stats.ox.ac.uk/genetics_software/shapeit/shapeit.html)<br>IMPUTE2 v2.3.2 (https://mathgen.stats.ox.ac.uk/impute/impute_v2.html)<br>Michigan Imputation Server v1.2.4 (https://imputationserver.sph.umich.edu)<br>GWAMA (https://genomics.ut.ee/en/tools)<br>STATA 10.1 (https://www.stata.com)<br>GCTA (https://yanglab.westlake.edu.cn/software/gcta)<br>LDSC v.1.0.1 (https://github.com/bulik/ldsc)<br>GWAS-PW (https://github.com/joepickrell/gwas-pw)<br>MIRTK toolkit (http://mirtk.github.io)<br>BOLT-LMM v2.3.2 (https://alkesgroup.broadinstitute.org/BOLT-LMM)<br>FUMA 1.5.1 (https://fuma.ctglab.nl)<br>MTAG v.1.0.8 (https://github.com/JonJala/mtag)<br>sc-linker (https://github.com/karthikj89/scgenetics)<br>OpenTargets, release of October 12th, 2022 (https://www.opentargets.org)<br>REGENIE (https://rgcgithub.github.io/regenie/)

MetaXcan (https://github.com/hakyimlab/MetaXcan)
PrediXcan (https://github.com/hakyimlab/PrediXcan)
MR-base v.0.5.6 (https://www.mrbase.org)
R project v.4.2.0 (https://www.r-project.org)

For manuscripts utilizing custom algorithms or software that are central to the research but not yet described in published literature, software must be made available to editors and reviewers. We strongly encourage code deposition in a community repository (e.g. GitHub). See the Nature Portfolio guidelines for submitting code & software for further information.

Data

Policy information about availability of data

All manuscripts must include a data availability statement. This statement should provide the following information, where applicable:

- Accession codes, unique identifiers, or web links for publicly available datasets
- A description of any restrictions on data availability
- For clinical datasets or third party data, please ensure that the statement adheres to our policy

Data from the Genome Aggregation Database (gnomAD, v.2.1.1) are available at https://gnomad.broadinstitute.org. Data from the UKB can be requested from the UKB Access Management System (https://bbams.ndph.ox.ac.uk). Data from the GTEx consortium are available at the GTEx portal (https://gtexportal.org). Published snRNA-seq data are available at the Broad Single Cell Portal (https://singlecell.broadinstitute.org/) and at the Cellxgene tool website (https://cellxgene.cziscience.com/collections/e75342a8-0f3b-4ec5-8ee1-245a23e0f7cb/private). The Genome assembly GRCh37 can be accessed using https://www.ncbi.nlm.nih.gov/datasets/genome/GCF_000001405.13/. Individual level data sharing is subject to restrictions imposed by patient consent and local ethics review boards. Full GWAS summary statistics of HCM, HCMSARC-, HCMSARC+, MTAG, and 10 LV traits are available on the GWAS catalog (accession IDs GCST90435254 to GCST90435267) and can be accessed interactively at www.well.ox.ac.uk/hcm.

Human research participants

Policy information about studies involving human research participants and Sex and Gender in Research.

Reporting on sex and gender

Biological sex is reported for all included study cohorts. Females constitute 31% of cases and 36% of controls in the HCM case-control GWAS, and 54% of UK Biobank participants with available cardiac MRI included in the left ventricular GWAS. Sex was included as a covariate in regression analyses. No sex-stratified analyses were performed.

Population characteristics

HCM GWAS cohort: mean age ranging from 51 to 67 across cohorts, median maximal left ventricular wall thickness 19mm, 32% carrying a presumably causal rare sarcomeric gene variant, 28% with obstructive physiology. Details are presented in Supplementary Table 1.

UK Biobank cardiac magnetic resonance cohort: mean age 63, mean body mass index 26, 25% with a diagnosis of hypertension. Details are presented in Supplementary Table 7.

Recruitment

Cases clinically diagnosed with HCM were enrolled from local, national or international studies. Controls without known HCM were included from population-matched cohorts. Details appear in the Supplementary Note.

Ethics oversight

All components of the study were approved by ethics review boards at corresponding institutions:
- Medisch Ethische Toetsingscommissie (METC) of the Amsterdam University Medical
- Hammersmith & Queen Charlotte’s Research Ethics Committee
- South Central - Hampshire B Research Ethics Committee
- Research Ethics and New Technology Development Committee of the Montreal Heart Institute
- South Central - Oxford A Research Ethics Committee
- East of England - Cambridge South Research Ethics Committee

Note that full information on the approval of the study protocol must also be provided in the manuscript.

Field-specific reporting

Please select the one below that is the best fit for your research. If you are not sure, read the appropriate sections before making your selection.

☒ Life sciences ☐ Behavioural & social sciences ☐ Ecological, evolutionary & environmental sciences

For a reference copy of the document with all sections, see nature.com/documents/nr-reporting-summary-flat.pdf

Life sciences study design

All studies must disclose on these points even when the disclosure is negative.

Sample size

For hypertrophic cardiomyopathy (HCM), we analyzed the largest sample size available, including 5,900 HCM cases and 68,359 controls. For left ventricular (LV) traits, we analyzed the largest sample size with available cardiac magnetic resonance imaging studies from the UK Biobank at the time of analysis, consisting of 36,083 individuals. For association of rare loss of function variants with HCM, we included 1,845 HCM

	cases and 37,481 controls. No method was applied to predetermine sample size. Such sample size allowed the identification of significant associations for ALPK3 and SVIL but may have been underpowered for genes with smaller effect sizes.
Data exclusions	Pre-established quality control processes were applied during inclusion (clinical and imaging data) and during analysis (genotypic data).
Replication	No replication of GWAS signals was attempted in the absence of a sufficiently powered independent cohort and considering the conventional significance threshold for GWAS set to P≤5e-8 in the meta-analysis.
Randomization	The GWAS used a case-control study design. In the process of selecting appropriate controls, the R package dplyr::sample_n was used to pseudo-randomly select controls.
Blinding	Blinding was not possible in the analysis plan.

Reporting for specific materials, systems and methods

We require information from authors about some types of materials, experimental systems and methods used in many studies. Here, indicate whether each material, system or method listed is relevant to your study. If you are not sure if a list item applies to your research, read the appropriate section before selecting a response.

Materials & experimental systems

n/a	Involved in the study	
☒	☐	Antibodies
☒	☐	Eukaryotic cell lines
☒	☐	Palaeontology and archaeology
☒	☐	Animals and other organisms
☒	☐	Clinical data
☒	☐	Dual use research of concern

Methods

n/a	Involved in the study	
☒	☐	ChIP-seq
☒	☐	Flow cytometry
☒	☐	MRI-based neuroimaging
